# Retrospective analysis of clinical features in 134 coronavirus disease 2019 cases

**DOI:** 10.1017/S0950268820002010

**Published:** 2020-09-03

**Authors:** Lin Zhang, Bin Huang, Hongzhen Xia, Hua Fan, Muxin Zhu, Liping Zhu, Huan Zhang, Xiaogen Tao, Shaohui Cheng, Jian Chen

**Affiliations:** 1 Department of Intensive Care Medicine, The First Affiliated Hospital of USTC, Division of Life Science and Medicine, University of Science and Technology of China, Hefei, Anhui, 230036, China; 2 Department of Endocrinology, The First Affiliated Hospital of USTC, Division of Life Science and Medicine, University of Science and Technology of China, Hefei, Anhui, 230036, China; 3 Department of Pathology, The First Affiliated Hospital of USTC, Division of Life Science and Medicine, University of Science and Technology of China, Hefei, Anhui, 230036, China; 4 Department of Intensive Care Medicine, Wuhan Jinyintan Hospital, Wuhan, Hubei, 430023, China

**Keywords:** Blood group distribution, coagulation abnormality, COVID-19, inflammatory factors, organ function injury

## Abstract

We aimed to describe the clinical features in coronavirus disease 2019 (COVID-19) cases. We studied 134 critically ill COVID-19 cases from 30 December 2019 to 20 February 2020 in an intensive care unit (ICU) at Wuhan Jinyintan Hospital. Demographics, underlying diseases, therapy strategies and test results were collected and analysed from patients on admission, admission to the ICU and 48 h before death. The non-survivors were older (65.46 (s.d. 9.74) *vs.* 46.45 (s.d. 11.09)) and were more likely to have underlying diseases. The blood group distribution of the COVID-19 cases differed from that of the Han population in Wuhan, with type A being 43.85%; type B, 26.92%; type AB, 10% and type O, 19.23%. Non-survivors tend to develop more severe lymphopaenia, with higher C-reactive protein, interleukin-6, procalcitonin, D-dimer levels and gradually increased with time. The clinical manifestations were non-specific. Compared with survivors, non-survivors more likely to have organ function injury, and to receive mechanical ventilation, either invasively or noninvasively. Multiple organ failure and secondary bacterial infection in the later period is worthy of attention.

## Introduction

In December 2019, a novel coronavirus emerged in Wuhan and was identified as the causal pathogen of coronavirus disease 2019 (COVID-19) [1]. COVID-19 is a pandemic currently affecting more than 3 million people worldwide and the rate of infection was rapid; the case fatality rate has exceeded 7% as of early May 2020 [2]. Current studies [3, 4] have demonstrated that COVID-19 often occur in elderly men with underlying diseases. Fatal respiratory distress syndrome and multiple organ failures are seen in the advanced and late stages of the disease.

However, there have been few studies on the clinical characteristics of COVID-19 death cases. To understand these clinical characteristics, a retrospective analysis of clinical features in 134 critically ill COVID-19 cases in Wuhan Jinyintan Hospital was carried out.

## Methods

### Study population

This study collected COVID-19 cases from 30 December 2019 to 20 February 2020 in an intensive care unit (ICU) at the Wuhan Jinyintan Hospital. The hospital specialises in infectious diseases and is prescribed by the Chinese government as one of the first designated treatment units for patients with the disease. The diagnosis of confirmed and clinical cases was made following the Diagnosis and Treatment of Novel Coronavirus Pneumonia (trial version 5) [5]. Confirmed cases were defined as cases that had pathogenic evidence, positive reverse transcription polymerase chain reaction, or highly homologous gene sequencing with known coronaviruses. Clinical cases were defined as cases that had epidemic history, or clinical symptoms and imaging characteristics. We defined epidemic history as suspected cases that had a travel history in Wuhan and surrounding areas within 14 days, exposure to other patients with COVID-19, communicated with patients with fever or respiratory symptoms from Wuhan and surrounding areas or from case-reporting communities, or cluster cases**.** Clinical features included fever or respiratory symptoms and decreased lymphocyte or leucocyte count in the early stage.

This study was approved by the Ethics Committee of Wuhan Jinyintan Hospital (KY-2020-28.01). The subjects participating in the study (or their relatives in this case) were the ones not required to give consent due to the rapid emergence of this infectious disease.

### Data collection

This retrospective analysis was based on case reports, nursing records and test results. The patients were categorised into the survivors and non-survivors group. The non-survivors' data were collected on admission, admission to the ICU and 48 h before death and the survivors' data were collected at admission. Data include demographics, underlying diseases, therapy strategies and test results of patients. The therapy strategy represents antiviral and antibacterial treatments, corticosteroid treatment, immunotherapy and respiratory therapy. Two experienced clinicians reviewed and summarised the data.

### Statistical analysis

SPSS (version 24.0) was used for all analyses and statistical significance was set at *P* < 0.05. Continuous measurements, such as the mean (s.d.), were utilised if data were normally distributed; however, if the data were not normally distributed, the median (interquartile range (IQR)) was utilised. Categorical variables were described utilising frequency and percentages. Independent tests, including the *t*-test, *χ*^2^ test or Mann–Whitney *U* test, were used to compare the survivors and non-survivors group. We compared the differences in laboratory measures among patients who died of COVID-19 on admission, admission to ICU and 48 h before deaths. Statistical analysis was performed by one-way analysis of variance.

## Results

### Demographics

Among the 134 patients, 87 were men and 47 were women, with an average age of 60.78 ± 12.98 years, ranging from 24 to 83 years. Many patients had underlying comorbidities with cardiovascular and cerebrovascular disease (39.55%) and endocrine system diseases (19.40%). Compared with survivors, the non-survivors were older (65.46 ± 9.74 *vs.* 46.23 ± 12.01) and were more likely to have chronic medical illnesses. The blood type distribution of COVID-19 patients was significantly different from that of the healthy Han population in Wuhan (43.85% type A, 26.92% type B, 10% type AB, 19.23% type O in COVID-19 patients; 32.16% type A, 24.91% type B, 9.10% type AB and 33.84% type O in the healthy Han population in Wuhan). However, the distribution of ABO blood type was not significantly different between survivors and non-survivors. The median time from symptom onset to hospital was 9.5 days (IQR 7–10.25) and 11 days (IQR 8.00–13.50) in the survivors and non-survivors, respectively (Tables 1 and 2).

**Table 1.** Demographics of 101 patients with COVID-19

	All patients（*n* = 134）	Survivors (*n* = 33)	Non-survivors (*n* = 101)	*P* value
Age, years				
Mean (s.d.)	60.78 (12.98)	46.45 (11.09)	65.46 (9.74)	0.00
Range	24–83	26–79	24–83	
≤44	19 (14.18%)	16 (48.48%)	3 (3.00%)	
45–59	24 (25.37%)	15 (45.45%)	19 (18.80%)	
60–74	61 (45.52%)	1 (3.03%)	60 (59.40%)	
≥75	20 (14.93%)	1 (3.03%)	19 (18.80%)	
Sex				
Female	47 (35.07%)	10 (30.30%)	37 (37.00%)	0.51
Male	87 (64.93%)	23 (69.70%)	64 (64.00%)	
Days from onset to admission	10.00 (8.00–13.00)	9.50 (7–10.25)	11.00 (8.00–13.50)	0.06
Blood group				
A	57 (43.85%)	13 (41.94%)	44 (44.44%)	0.45
B	35 (26.92%)	6 (19.35%)	29 (29.29%)	
AB	13 (10%)	5 (16.13%)	8 (8.08%)	
O	25 (19.23%)	7 (22.58%)	18 (18.18%)	
Chronic medical illness				
Cardiovascular and cerebrovascular disease	53 (39.55%)	4 (12.12%)	49 (48.51%)	0.00
Endocrine system disease	26 (19.40%)	3 (9.09%)	23 (22.77%)	0.13

Values are median (IQR) or *n*/*N* (%), where *N* is the total number of patients with available data. *P* values comparing survivors and non-survivors are from *t*-test, *χ*^2^ test or Mann–Whitney *U* test. COVID-19 = coronavirus disease 2019.

**Table 2.** Comparison of ABO blood type distribution between COVID-19 patients and healthy Han population in Wuhan

Blood type	A	B	AB	O	
Control	1188 (32.16%)	920 (24.91%)	336 (9.10%)	1250 (33.84%)	3694 (100%)
COVID-19	57 (43.85%)	35 (26.92%)	13 (10%)	25 (19.23%)	130 (100%)
*c*^2^	4.865	0.314	0.004	11.336	12.153
*P* value	0.027	0.575	0.949	0.001	0.007

Values are *n*/*N* (%), where *N* is the total number of patients with available data.

### Clinical features

The relative frequencies of all reported symptoms at the time of admission are shown in Table 3. The most common symptom was fever (*n* = 121, 90.30% of 134 patients), but most patients presented normal temperature after 1–3 days of admission, which may be related to glucocorticoids use. Cough (*n* = 94 (70.15%)) and dyspnoea (*n* = 84 (64.93%)) were also common. Additionally, 42 (31.34%) patients had white sputum at the early stage, and five patients showed yellowish purulent sputum. Other common symptoms included myalgia, general weakness, dizziness, headache, nausea and vomiting. Distinctive from severe acute respiratory syndrome (SARS), only two patients with COVID-19 had diarrhoea. Compared with the survivors, non-survivors were more likely to report dyspnoea. Most patients had complications, included ARDS (105 (78.36%) patients), followed by cardiac injury (75 (55.97%) patients), acute kidney injury (63 (47.01%) patients) and liver injury (53 (39.55%) patients) (Table 3).

**Table 3.** Clinical characteristics, treatment and complications of patients with COVID-19

	All patients (*n* = 134)	Survivors (*n* = 33)	Non-survivors (*n* = 101)	*P* value
Symptoms at admission				
Fever	121 (90.30%)	30 (90.91%)	91 (90.10%)	0.99
Highest temperature, °C				
<37.3	13 (9.70%)	3 (9.09%)	10 (9.90%)	0.50
37.3–38	40 (29.85%)	7 (23.33%)	33 (32.67%)	
38.1–39	62 (46.27%)	20 (60.61%)	42 (41.58%)	
>39	19 (14.18%)	3 (9.09%)	16 (15.84%)	
Non-productive cough	47 (35.07%)	9 (27.27%)	38 (37.62%)	0.30
Productive cough	47 (35.07%)	16 (48.48%)	31 (30.69%)	0.09
Haemoptysis	3 (2.24%)	0 (0%)	3 (2.97%)	0.99
Sore throat	6 (4.48%)	0 (0%)	6 (5.94%)	0.34
Dyspnoea	87 (64.93%)	12 (36.36%)	75 (74.26%)	0.00
Chills	22 (16.42%)	9 (27.27%)	13 (12.87%)	0.06
Myalgia	14 (10.45%)	5 (15.15%)	9 (8.91%)	0.33
Diarrhoea	2 (1.49%)	0 (0%)	2 (1.98%)	0.99
Malaise	31 (23.13%)	10 (30.30%)	21 (20.79%)	0.34
Nausea or vomiting	9 (6.72%)	2 (6.06%)	7 (6.93%)	0.99
Dizziness	9 (6.72%)	2 (6.06%)	7 (6.93%)	0.99
Headache	6 (4.48%)	3 (9.09%)	3 (2.97%)	0.14
Treatment				
Invasive mechanical ventilation	79 (58.96%)	0 (0%)	79 (78.22%)	0.00
Non-invasive ventilation or high-flow nasal cannula	91 (6.79%)	7 (21.21%)	84 (83.17%)	0.00
ECMO	7 (5.22%)	0 (0%)	7 (6.93%)	0.19
CRRT	8 (5.97%)	0 (0%)	8 (7.92%)	0.19
Antiviral treatment	86 (64.18%)	25 (75.76%)	61 (60.40%)	0.14
Antibiotic treatment	131 (97.76%)	30 (90.91%)	101 (100%)	0.01
Antifungal treatment	24 (17.91%)	1 (3.03%)	23 (22.77%)	0.00
Glucocorticoids	64 (47.76%)	5 (15.15%)	59 (58.42%)	0.00
Intravenous immunoglobulin therapy	69 (51.49%)	5 (15.15%)	64 (63.37%)	0.00
Thymosin	47 (35.07%)	2 (6.06%)	45 (44.55%)	0.00
Complications				
ARDS	105 (78.36%)	5 (15.15%)	100 (99.01%)	0.00
Acute cardiac injury^a^	75 (55.97%)	2 (6.06%)	73 (72.27%)	0.00
Pneumothorax	3 (2.24%)	0	3 (2.97%)	0.99
Liver dysfunction	53 (39.55%)	9 (27.27%)	44 (43.56%)	0.10
Acute kidney injury	63 (47.01%)	1 (3.03%)	62 (61.39%)	0.00

CRRT, continuous renal replacement therapy; ECMO, extracorporeal membrane oxygenation.

Values are *n*/*N* (%), where *N* is the total number of patients with available data.

a Defined as blood levels of cardiac biomarkers (hs-Troponin I (hs-TnI) above the 99th percentile of the upper reference limit, regardless of new abnormalities in electrocardiography and echocardiography.

### Laboratory findings

**Fig. 1.**
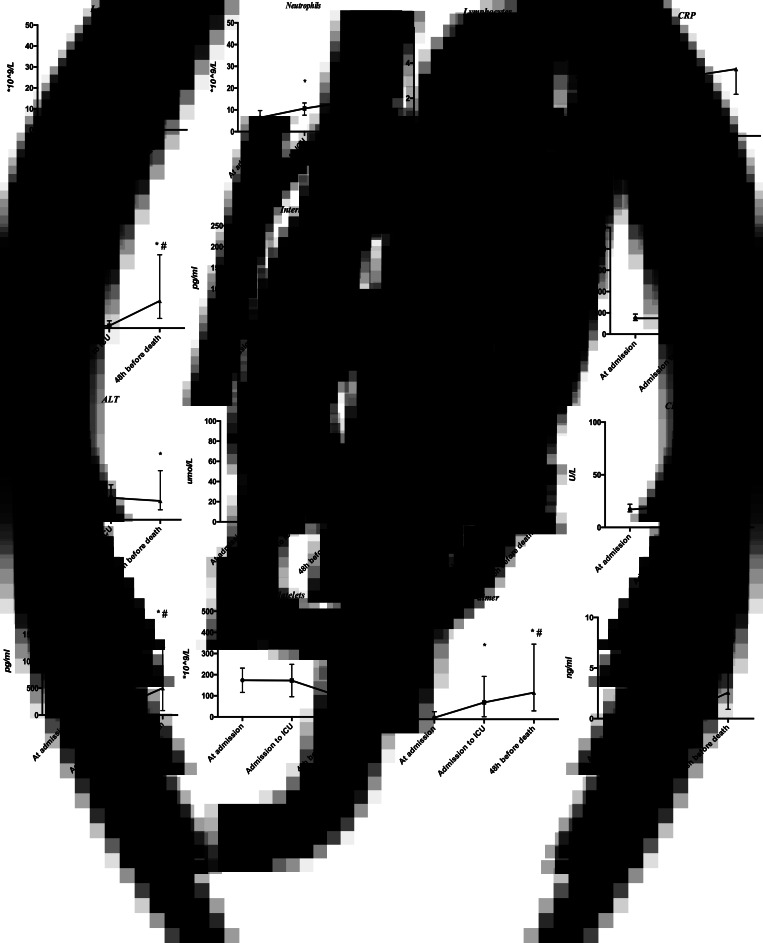
Dynamic profile of laboratory parameters in 101 patients with COVID-19. Timeline charts illustrate the laboratory parameters in 101 patients with COVID-19 when their entrance into admission, ICU and 48 h before death. *P < 0.05 *vs.* at admission group, ^#^*P* < 0.05 *vs.* admission to ICU group.

On admission, most patients had marked lymphopaenia, but non-survivors tend to develop more severe lymphopaenia. Inflammatory indicators such as leucocytes, neutrophils, C-reactive protein (CRP), procalcitonin (PCT) and interleukin-6 (IL-6) levels were higher in non-survivors than in survivors and gradually increased with time. It was found, through dynamic analysis of coagulation-related indicators, that as platelets (PLT) counts decreased, D-dimer and prothrombin time (PT) increased correspondingly during the disease progression. The level of hs-TnI was 19.9 (8.98–79.65) pg/ml in non-survivors, which was higher than that of the survivors. Owing to the progress of the disease, myocardial damage indicators were significantly increased. Liver and kidney injuries were not significant on admission, but as the disease progressed, the levels of blood urea and creatinine progressively increased before death. Experimental data are given in Table 4 and Figure 1.

**Table 4.** Laboratory findings of patients with COVID-19

	Survivors (*n* = 33)	Non-survivors (*n* = 101)	*P* value
Leucocytes ( × 10⁹/l; normal range 3. 5–9.5 × 10⁹/l)	6.48 (4.98–10.07)	9.08 (6.18–12.47)	0.014
Neutrophils ( × 10⁹/l; normal range 1.8–6.3 × 10⁹/l)	4.99 (2.72–7.78)	8.18 (5.08–11.78)	0.001
Lymphocytes ( × 10⁹/l; normal range 1.1–3.2 × 10⁹/l)	1.00 (0.69–1.28)	0.55 (0.39–0.72)	0.000
PLT ( × 10⁹/l; normal range 125.0–350.0 × 10⁹/l)	206 (151.75–278.25)	172 (123–220)	0.016
PT time (s; normal range 10.5–13.5 s)	10.9 (10.23–11.75)	11.90 (11.10–13.00)	0.000
D-dimer (μg/l; normal range 0.0–1.5 μg/l)	0.54 (0.35–1.14)	3.15 (1.06–18.87)	0.000
ALT (U/l; normal range 7–40 U/l)	28 (20–43)	40.0 (21–63.5)	0.130
AST (U/L; normal range 13–35 U/l)	32 (25–43)	47 (34.5–66.5)	0.000
Indirect bilirubin (μmol/l; normal range 0–17 μmol/l)	8.1 (5.95–9.65)	8.9 (6.75–12.85)	0.067
Serum creatinine (μmol/l; normal range 57–111 μmol/l)	75.2 (59.25–84.40)	73.2 (62.55–95.85)	0.391
Blood urea nitrogen (mmol/l; normal range 3.6–9.5 mmol/l)	4.60 (3.45–6.15)	7.3 (5.20–9.00)	0.000
Albumin (g/l; normal range 35–55 g/l)	33.7 (30.9–36.15)	28.6 (25.8–30.7)	0.000
hsTroponin I (U/l; normal range 0–28 U/l)	3.4 (1.75–4.50)	19.9 (8.98–79.65)	0.000
CK-MB (U/l; normal range 0–24 U/l)	15 (13.0–19.50)	18.5 (15.0–24.0)	0.004
LDH (U/l; normal range 120–250 U/l)	277 (215.5–320)	504.5 (381.25–644.25)	0.000
Myoglobin (ng/ml; normal range 0.0–146.9 ng/ml)	47 (27.9–98.4)	109.5 (57.5–204)	0.000
CRP (mg/l; normal range 0.0–5.0 mg/l)	43.3 (20.5–74.60)	121.55 (69.08–160.0)	0.000
PCT (ng/ml; normal range <0.5 ng/ml)	0.05 (0.05–0.093)	0.12 (0.05–0.33)	0.000
IL-6 (pg/ml; normal range 0.0–7.0 pg/ml)	5.11 (3.92–6.09)	9.15 (6.90–14.05)	0.000

COVID-19, coronavirus disease 2019; CK-MB, creatinine kinase-MB; AST, aspartate aminotransferase.

Values are median (IQR) unless stated otherwise.

### Treatments

All patients were treated in isolated wards. Among them, 86 (64.18%) received antiviral drugs, including Oseltamivir, Ribavirin, Lopinavir, Ritonavir, Ganciclovir, Interferon, etc. Glucocorticoids, intravenous immunoglobulins and thymosin preparations were used in 64 (47.76%), 69 (51.49%) and 47 (35.07%) patients, respectively. The proportion of patients receiving antibiotic agents was 97.76%. Antibiotic use was generally broad in spectrum with fluoroquinolones and carbapenems. Twenty-three (22.78%) non-survivors and one (3.03%) survivor received antifungal drugs.

Compared with survivors, non-survivors were more likely to develop ARDS, and to receive mechanical ventilation. Eighty-four (83.17%) non-survivors were treated with non-invasive ventilator or high-flow nasal cannula oxygen, and 79 (78.22%) non-survivors were treated with invasive mechanical ventilation. The median time from ARDS to invasive mechanical ventilation was 3 days (IQR 0.00–6.00), of which 21 non-survivors were intubated 2 days before death. The duration of invasive mechanical ventilation was 1–31 days (median 5 days (IQR 2.00–8.00)). Seven non-survivors were treated with extracorporeal membrane oxygenation and eight with continuous renal replacement therapy (Table 3)

## Discussion

The present analyses revealed the clinical characteristics of 134 cases caused by COVID-19 in China. Among the 134 patients, males dominated and the proportion of elderly patients with underlying diseases was relatively high. Importantly, we found that the blood group distribution of the COVID-19 patients (A: 43.85%, B: 26.92%, O: 19.23%, AB: 10%) differed from that of the Han population in Wuhan [6]. Type O was relatively low, but type A was relatively high. However, the distribution of ABO blood type was not significantly different between survivors and non-survivors. ABO blood group antigen substances are widely distributed in the human respiratory, digestive tract and reproductive systems [7]. Previous studies have shown that ABO blood groups are related to the onset and spread of various diseases because the blood group antigens may be involved in virus infection as receptors [8, 9]. In the study of various susceptible genes in SARS-CoV, individuals in the blood group O had a lower infection rate [10]. Guillon *et al*. found that type A antibodies can provide protection by inhibiting interaction between the virus and ACE2 receptor [11]. However, the higher proportion of patients with type A blood remains unclear although the lack of antibody A protection might be involved. Further research is needed to explore the mechanism by which the patients of type A are more susceptible to COVID-19 infection.

The clinical manifestations of COVID-19 are non-specific, which is consistent with previous studies [12], and the most common symptom is fever. However, not all patients had fever; for instance, no fever was noted in 13 (9.70%) patients at the onset of the disease. Moreover, 22 (16.42%) patients had no respiratory symptoms at the beginning of the disease, and fever and chest tightness gradually occurred as the disease progressed. Therefore, the delay of fever and respiratory symptoms may affect the early identification of COVID-19.

The mortality of critically ill COVID-19 patients is high, but its mechanism is not clear at present; it may be related to the virus-induced acute lung injury, inflammatory factor storm, multiple organ damage and nosocomial infections. We collected laboratory examination results on admission, at the time of transfer to the ICU, and at 48 h before death, and found that CRP and IL-6 levels to be higher in non-survivors than survivors. The level of IL-6 gradually increased as the disease progressed in non-survivors and was up to 26.21 pg/ml (IQR 11.68–205.92) 48 h before death, which was significantly higher than results reported by Chen *et al*. [3]. This indicates that there was a severe inflammatory reaction. We found that 23 (22.78%) non-survivors had abnormal coagulation function at admission, which mainly manifested as increased D-dimer levels and a sudden deterioration. Under these circumstances, attention should be paid to the presence of pulmonary thromboembolism after micro-thrombosis in the lungs or deep vein thrombosis. For hypercoagulable patients without contraindications, reasonable anticoagulant therapy is a possible choice.

Additionally, our study discovered that as the disease progressed, leucocytes and neutrophils counts, along with PCT and CRP levels gradually increased; in conjunction, we noticed lymphocyte levels increasing after the initial decline. Additionally, some patients experienced a drop in body temperature and then increased or continued fever fluctuations. These indicators do not fully meet the characteristics of viral infections. It is necessary to be alert to those patients who may have secondary bacterial *infection*. In a recent systematic review and meta-analysis that included 28 studies evaluating co-infections and secondary bacterial infection among COVID-19 cases, the authors reported bacterial infection ranged from 5.8% in all hospitalised patients to 8.1% in critically ill patients and 11.6% in fatal cases [13]. Secondary bacterial and fungal infections may be related to suppressed immunity, lack of medical resources and unsmooth sputum drainage. In addition, clinical manifestations of bacterial or fungal infections in critical patients may be inconspicuous owing to their compromised immunity or glucocorticoids use. Therefore, it is essential to keep monitoring temperature, laboratory indicators, imaging indicators and airway secretion characteristics of the patients. Prevention and control of secondary bacterial infections are also needed.

In terms of treatment, the use of antiviral drugs and glucocorticoids is still controversial. In this study, 64.18% of patients received antiviral drugs, 47.76% patients were given glucocorticoids and the treatment course was mostly 3–5 days. Most patients received antibiotic treatments including fluoroquinolones and carbapenems. But there is currently insufficient evidence to support widespread use of antibiotics in most hospitalised patients. Mechanical ventilation is the main supportive treatment for critically ill patients, but the overall survival time of patients after invasive mechanical ventilation was short (median 5 days (IQR 2.00–8.00)). Most patients did not benefit significantly from invasive mechanical ventilation and had suffered multiple organ failure caused by severe hypoxia before invasive mechanical ventilation. Therefore, for critical patients, early invasive mechanical ventilation treatment should be in consideration.

This study has some limitations. The sample size can be increased in further research for prospective case-control study. The blood group composition of patients cannot be statistically analysed, and whether the blood group difference is related to the susceptibility of COVID-19 infection needs to be clarified.

## Conclusion

Critical COVID-19 may cause fatal respiratory distress syndrome and multiple organ failure with a high case fatality rate. The blood type distribution of COVID-19 patients was different from that of the healthy Han population in Wuhan. But the distribution of ABO blood types was not significantly different between survivors and non-survivors. Multiple organ failure and secondary bacterial infection in the later period is worthy of attention.

## Data Availability

After publication, the data will be made available to others on reasonable requests to the corresponding author. Deidentified participant data will be provided after approval from the corresponding author, Wuhan Jin Yin-tan Hospital and The First Affiliated Hospital of USTC.
